# Death depression in Egyptian clinical and non‐clinical groups

**DOI:** 10.1002/nop2.601

**Published:** 2020-08-31

**Authors:** Ahmed M. Abdel‐Khalek, Mahboubeh Dadfar, David Lester

**Affiliations:** ^1^ Department of Psychology Faculty of Arts Alexandria University Alexandria Egypt; ^2^ School of Behavioral Sciences and Mental Health‐Tehran Institute of Psychiatry International Campus School of Public Health, Student Committee of Education and Development Center (EDC) Iran University of Medical Sciences Tehran Iran; ^3^ Psychology Program Stockton University Stockton NJ USA

**Keywords:** addicts, anxious outpatients, death depression, gender‐related differences, non‐clinical group, schizophrenics

## Abstract

**Aim:**

The main aims of this study were to explore the differences between seven Egyptian clinical and non‐clinical samples in death depression, as well as to estimate gender‐related differences.

**Design:**

A cross‐sectional study.

**Methods:**

The Death Depression Scale (DDS) was administered to seven groups (*N* = 765) of Egyptian normal (non‐clinical) patients, anxiety outpatients, schizophrenic inpatients (men and women) and addicts (men only) in individual sessions.

**Results:**

Anxiety outpatients of both sexes obtained significantly and greatly higher death depression scores than did the other five groups, whereas the male schizophrenics, the male addicts, and the male and female non‐clinical groups had the lowest death depression scores. Female schizophrenics obtained a significantly higher death depression scores than did male schizophrenics, addicts and non‐clinical participants. Female anxiety outpatients and schizophrenics had higher death depression mean scores than did their male counterparts.

**Discussion:**

The present finding is consistent, in general, with previous studies on death anxiety and death obsession. What applied to death anxiety was consistent also with death depression and death obsession. That is, the death distress concept.

## 1 BACKGROUND

Most people probably notice that we feel blue, become sad, and depressed about death, both our own death and the death of significant others. Therefore, researchers have explored the existence of depression regarding death (Temple, Lavoie, Chalgujian, & Thomas‐Dobson, 1990). Drawing on her impressions of terminal cancer patients, Kübler‐Ross (1969) stated that depression is the fourth stage of the dying process. Erikson (1959) maintained that, in the final stage of life, persons without a sense of ego integrity view themselves as failures and suffer from despair. Research has demonstrated a close relationship between death, depression and bereavement (Parkes, 1986, p. 128f; Raphael, 1984; Stroebe, Stroebe, & Hansson, 1993). Basing their argument on clinical work, Schultz and Aderman (1974) also emphasized a strong element of depression in the dying process. Existential philosophers such as Heidegger (1962), Kierkegaard (1981) and Sartre (1956) have addressed a similar phenomenon, and written on despair and a sense of meaninglessness or absurdity in connection with the finiteness of life. At the conceptual and psychological level, Templer et al. (1990) introduced the concept and devised a scale of death depression, and published a number of research papers (Alvarado, Templer, Bresler, & Thomas‐Dobson, 1993, 1995; Hintze, Templer, Cappelletty, & Frederick, 1993; Siscoe, Reimer, Yanovsky, Thomas‐Dobson, & Templer, 1992).

## 2 PROBLEM IDENTIFICATION

The vast majority of humans have negative attitudes towards death. Within these attitudes, the affective aspect is a salient component. Foremost among this affective component is the negative emotions of anxiety, depression and obsession (Al‐Sabwah and Abdel‐Khalek, 2006b; Bahrami, Dadfar, Lester, & Abdel‐Khalek, 2014; Dadfar & Lester, 2014, 2015; Dadfar, Abdel‐Khalek, & Lester, 2017; Dadfar, Asgharnejad Farid, Lester, Atef Vahid, & Birashk, 2016; Dadfar, Lester, Abdel‐Khalek, & Ron, 2018; Dadfar, Lester, Atef Vahid, Asgharnejad Farid, & Birashk, 2014; Macedo, 2019; Schumaker, Warren, & Groth‐Marnat, 1991). In two previous studies, Abdel‐Khalek (2002a, 2005a) examined death anxiety and death obsession in seven clinical and non‐clinical samples.

Using three different Arab college students' samples from Egypt, Kuwait and Lebanon, Abdel‐Khalek (1997, 1998, 2001) administered the Death Depression Scale (DDS), the Death Anxiety Scale (DAS), the Trait Anxiety Scale (TAS) and the Beck Depression Inventory (BDI). He found that DDS scores were correlated significantly with DAS and TAS scores in these three samples, and with BDI scores in two out of the three samples. In all samples, a principal component analysis followed with a Varimax orthogonal rotation extracted two components labelled: Death Distress and General Neurotic Disorders.

In another study, Abdel‐Khalek (2004a) administered the Arabic Scale of Death Anxiety (ASDA), the DDS and the Death Obsession Scale (DOS) to seven Egyptian clinical and non‐clinical groups. The results indicated that the correlations between the scales were statistically significant and positive. A general factor of Death Distress was extracted in all seven groups. Abdel‐Khalek (2004c) reported that ASDA scores correlated with DDS, DOS and Reasons for Death Fear Scale (RDFS) scores in three Egyptian, Kuwaiti and Syrian samples. Abdel‐Khalek (2012) also found significant associations between DDS, DAS and DOS scores, and he constructed a death distress short scale based on these scales.

A sample of 275 Arab undergraduates responded to the Oxford Happiness Inventory (OHI), the DAS, the ASDA, the DDS and the DOS. The results indicated that all correlations between OHI and the death distress scales cores were non‐significant, except one pertaining to happiness and DDS (negative) in women. Two oblique factors were extracted: Death Distress and Happiness (Abdel‐Khalek, 2005b).

Al‐Sabwah and Abdel‐Khalek (2006a) recruited a sample of Egyptian women studying nursing (*N* = 570). The aim was to explore the association between religiosity and death distress (using the DAS, ASDA, DDS and DOS). The results indicated significant and negative correlations between religiosity and the DAS, ASDA and DDS. A principal component analysis retained a single bipolar factor labelled Death Distress versus Religiosity. A sample of Arab college students (*N* = 245) responded to the Love of Life Scale (LLS), the DAS, ASDA, DDS and DOS (Abdel‐Khalek, 2007). All the correlations between the LLS and death distress scales were non‐significant, except one between LLS and DDS (negative) in women. Two oblique factors were extracted and labelled: Death Distress and Love of Life. It was concluded that these constructs represent two distinct and independent factors. Lester (2003) concluded that the DAS, the Collett‐Lester Fear of Death Scale (CLFDS), DDS and DOS measure different constructs.

The present study has two research questions as follows: (a) What is the difference between clinical and non‐clinical samples in death depression? (b) Are the gender differences in death depression significant? We hypothesized that (a) the differences in death depression between the seven groups would be significant, and (b) there will be gender‐related significant differences in death depression in favour of women.

## 3 METHODS

### 3.1 Participants

A convenience sample of 765 Egyptian participants took part in this study. They were classified into seven subgroups as follows: non‐clinical (normal) men and women, male and female anxiety disorder outpatients from a psychiatric clinic, male and female inpatients suffering from schizophrenia recruited from a psychiatric hospital, and a group of male addicts, inpatients in a special ward in a psychiatric hospital.

The non‐clinical groups stated that they had never been treated for neurotic or psychiatric difficulties. Thus, they were best described as non‐clinical groups. All of the anxiety disorder two groups were outpatients in psychiatric hospitals. They met the criteria for a current diagnosis of different subcategories of anxiety disorder according to the DSM‐IV American Psychiatric Association (APA, 2000) as applied by the psychiatrist using a psychiatric interview. The majority were diagnosed as GAD, whereas the minority were cases of panic, PTSD and specific phobia disorders. Regarding the inpatients suffering from schizophrenia, they were hospitalized in a governmental psychiatric hospital. All of them were diagnosed as suffering from schizophrenia in its broad definition. They contained the paranoid, disorganized, undifferentiated types of schizophrenia. The last group was the male addicts. Their abuse of, and dependence on drugs was diffused to a wide spectrum of substance and drugs, mainly cannabis. All of addicts were inpatients in separate departments for addiction treatment appended to a psychiatric hospital.

The inclusion criteria for each of the study groups were as follows: (a) age between 20–40 years, (b) occupation (employee or student) and (c) education (minimum nine years of education). The seven samples were generally matched as groups on the following variables: age, occupation and the years of schooling. Matching between groups was based on the last‐mentioned criteria as groups and not as individuals. In the anxiety disorder two groups, the outpatients which had other psychiatric comorbidities were excluded. There was no problem in the administration of the DDS to schizophrenic patients while taking their medication. Their answers to the DDS were reliable during hospitalization, because they were without of the exacerbated symptoms. They had not active hallucinations or delusions or formal thought disorders and their cooperation was good. The samples used in the present study were the same samples used in previous studies (Abdel‐Khalek, 2002a, 2004a, 2004b, 2005a).

### 3.2 Measures

The Death Depression Scale (DDS). The DDS was constructed and validated by Templer et al. (1990). It consists of 17 items with a true–false format. It has good internal consistency, face or content validity, a meaningful factorial structure and good construct validity. To develop the Arabic form, Abdel‐Khalek (1997) translated the English version into Arabic. This preliminary translation was carefully evaluated by two native speakers competent in both languages: a psychologist and a linguist to check the comparability of meaning. Suitable revisions and corrections were made. To estimate the cross‐language equivalence of the DDS, bilingual patients (*N* = 25) enrolled in the fourth year in the Department of English Language and Literature in Alexandria University, Egypt responded to the two forms. The correlation coefficient for the total score on the two forms was 0.864, indicating that the two forms of the scale functioned as equivalent stimuli (Addel‐Khalek, 1997). Cronbach *a* coefficients ranged from 0.83–0.89 in three Arab Egyptian, Kuwaiti and Lebanonese samples (Abdel‐Khalek, 2002a), indicating good internal consistency. Good psychometric properties have also been reported for the Farsi version of the DDS with Iranian samples (see Aghazadeh, Mohammadzadeh, & Rezaie, 2014; Dadfar & Lester, in press, 2016, 2017, 2018, 2020a, 2020b; Dadfar, Abdel‐Khalek, & Lester, 2018; Dadfar, Abdel‐Khalek, Lester, & Atef Vahid, 2017; Dadfar, Lester, & Abdel‐Khalek, 2018; Dadfar, Lester, Asgharnejad Farid, Atef Vahid, & Birashk, 2014, 2017; Mohammadzadeh, Rezaei, & Aghazadeh, 2016).

### 3.3 Procedure

The DDS, along with a battery of other personality scales, was administered anonymously and individually to each participant by a Ph.D. candidate. The battery of other personality scales included the ASDA, the Templer's DAS and the DOS, in addition to the DDS. All the participants were volunteers.

### 3.4 Data analysis

Descriptive statistics, *t* test, and one‐way analysis of variance were used (SPSS, 2009).

## 4 FINDINGS

Table 1 depicts the descriptive statistics for the samples. As can be seen in Table 1, the mean ages of the seven groups range between 31–32 years and the standard deviations are around 5.0. Therefore, the ages of the seven groups were closely similar.

**Table 1:** Means (*M*) and standard deviations (*SD*) of ages for the seven groups

	*N*	*M*	*SD*
Male normals	110	32.4	5.2
Female normals	110	31.5	5.9
Male anxiety patients	109	32.9	5.1
Female anxiety patients	109	31.7	5.6
Male schizophrenics	107	32.6	5.9
Female schizophrenic	110	32.1	5.2
Male addicts	110	32.1	5.3

Table 2 presents the descriptive statistics for the DDS among the seven groups. A one‐way analysis of variance for the seven groups on the DDS was computed. The *F* ratio (99.85) is highly statistically significant. The Scheffe procedure revealed that the two groups of female and male anxious patients had the highest mean scores and were significantly different from each other and from all the other five groups. On the other hand, the male schizophrenics, male addicts, and female and male normal patients had the lowest death depression mean scores. The female schizophrenics had significantly different DDS scores from all the other six groups. They had higher scores than the male schizophrenics, addicts, and normal participants, but a lower death depression score than did anxious outpatients.

**Table 2:** Mean (*M*) and standard deviation (*SD*) of the Death Depression Scale (DDS) in seven groups

Samples	*N*	DDS	
		*M* *	*SD*
Male normal patients	110	8.44_a_	3.53
Female normal patients	110	8.10_a_	2.22
Male anxiety patients	109	12.29_b_	2.00
Female anxiety patients	109	14.58_c_	1.91
Male schizophrenics	107	7.03_a_	3.61
Female schizophrenics	110	10.15_d_	2.55
Male addicts	110	7.33_a_	3.32
*F* ratio		99.85**	

* Scheffe procedure indicates that means sharing a common subscript do not differ at *p* < .05.

** *p* < .0001.

To test the hypothesis of gender differences in the DDS, the male addict group was excluded because there was no female addict group. Then, the differences between male versus female participants were computed. As shown in Table 3, all the differences were significant (except for the DDS in normal patients), that is, females obtained higher mean scores than did their male counterparts.

**Table 3:** Gender differences in Death Depression Scale (DDS)

Males versus. females	*df*	DDS	
		*M*.diff.	*t*
Normal patients	218	0.34	0.82
Anxiety disorder patients	216	2.29	8.27*
Schizophrenic patients	215	3.12	7.06*
All males versus females	653	1.69	5.63*

* *p* < .001.

## 5 DISCUSSION

The first hypothesis was fully verified. There were statistically significant differences between the seven clinical and non‐clinical groups. The salient result is the significantly and greatly higher death depression scores of the male and female anxiety outpatients in proportion to all the other five groups.

This result is congruent with the previously published reports on the comparison between the same seven groups in death anxiety and death obsession (see Abdel‐Khalek, 2002a, 2005a). In Abdel‐Khalek's (2002a) study on death obsession among the same groups, it was found that the most singular findings are that the two groups of anxiety disorder patients' total mean scores on death obsession were greatly and significantly higher than the total mean scores of the other five groups. Furthermore, the other salient differences on death obsession were that female schizophrenics had a significantly higher total mean scores on death obsession than both male normal (non‐clinical) and male schizophrenics. The male schizophrenics and male normals obtained the lowest mean score on death obsession, respectively. The male addicts obtained a total mean score on death obsession that was less than the two anxiety disorder patients. The majority of the afore‐mentioned results on death obsession is congruent with the present findings on death depression. These results may indicate the validity of the concept and factor of death distress (Abdel‐Khalek, 2004a, 2012). This finding may also support the significant associations between death attitudes (i.e. death anxiety, death obsession and death depression) and anxiety disorders. Furthermore, several studies have found significant associations between death distress and both general anxiety and neuroticism (Abdel‐Khalek, 1997, 1998, 2001, 2002b; Neimeyer & Van Brunt, 1995).

Male and female normal patients (non‐clinical), male schizophrenics and addicts obtained the lowest death depression scores. There were no significant differences between these four groups. However, the differences between the afore‐mentioned groups and both anxiety outpatients and female schizophrenics on the DDS were statistically significant. The low death depression mean scores among the non‐clinical group are predictable. On the other hand, the low death depression mean score of male addicts may reflect their indifference towards death. This result also may be discussed in relation to the substance abuse, the severity of addictive disorder and other psychiatric comorbidities which are very common in this population.

In a similar view, using the same seven groups, but the scale was the ASDA (Abdel‐Khalek, 2005a), it was found that total mean score in the female and male anxiety disorder patients, respectively, were significantly higher than the total mean scores of the other five groups. On the other hand, male schizophrenics obtained the lowest total mean score on death anxiety vis‐a‐vis all the other six groups, including the non‐clinical two groups. It was concluded in that previous study that the highest death anxiety scores are related to anxiety disorder ("neurosis" in the old none cluster) more than to psychosis and psychoticism.

As for the second hypothesis, the gender‐related differences in the death depression were statistically significant for the anxiety outpatients and schizophrenic inpatients, as well as the total group of men versus women, in which women had higher mean scores than did their male counterparts. This result is consistent with findings for death anxiety (Abdel‐Khalek, 1986; Dattell & Neimeyer, 1990; Kastenbaum, 2000; Templer, 1991), and for death depression and for death obsession in undergraduates (Abdel‐Khalek, 2012).

Despite the large number of participants, the seven different groups and the good psychometric properties of the DDS, some limitations have to be acknowledged. Foremost among them is the limited age range of the present samples. Because of the study design and sampling method, the results could have been confounded by many factors, for example the convenience nature of the sample selection and SES variables. In addition, a useful next step would be to use the Death Depression Scale‐Revised (DDS‐R) (Templer et al., 2002) in order to explore whether the use of the revised DDS scale confirms the present results.

## 6 CONCLUSION

The present finding is consistent, in general, with previous studies on death anxiety and death obsession. The new addition of the present study was as follows: what applied to death depression was consistent also with death anxiety and death obsession, supporting the death distress concept. The anxiety disorder outpatients obtained significantly the higher DDS than did the other five groups.

## CONFLICT OF INTERESTS

The authors declare that there is no funding source, and they have no conflict of interest regarding the publication of this paper.

## AUTHOR CONTRIBUTIONS

All authors have agreed on the final version and meet at least one of the following criteria [recommended by the ICMJE (http://www.icmje.org/recommendations/)]: substantial contributions to conception and design, acquisition of data or analysis and interpretation of data; and drafting the article or revising it critically for important intellectual content.

## ETHICS APPROVAL

This study was approved by the Research Ethics Committee of Alexandria University.

## Data Availability

The data are not publicly available due to the restrictions, for example their containing information that could compromise the privacy of research participants.
